# Pollination of Enclosed Avocado Trees by Blow Flies (Diptera: Calliphoridae) and a Hover Fly (Diptera: Syrphidae)

**DOI:** 10.3390/insects16090899

**Published:** 2025-08-27

**Authors:** David F. Cook, Muhammad S. Tufail, Elliot T. Howse, Sasha C. Voss, Jacinta Foley, Ben Norrish, Neil Delroy

**Affiliations:** 1 Department of Primary Industries and Regional Development, Horticulture and Irrigated Agriculture Directorate, 3 Baron-Hay Court, South Perth, WA 6151, Australia; shoaib.tufail@dpird.wa.gov.au (M.S.T.); elliot.howse@dpird.wa.gov.au (E.T.H.); 2 School of Biological Sciences, The University of Western Australia, 35 Stirling Highway, Crawley, WA 6009, Australia; sasha.voss@uwa.edu.au; 3 Jasper Farms Pty Ltd., Busselton, WA 6280, Australia; jacinta.f@jasperfarms.com.au (J.F.); bnorrish@delroy.com.au (B.N.); neil@delroy.me (N.D.)

**Keywords:** blowfly, drone fly, fly pollination, horticulture, avocado production, fruit set

## Abstract

Despite flies regularly visiting flowers, limited research has gone into their pollination ability on commercial crops. A national project in Australia aimed to identify fly species as potential managed pollinators for the horticultural industry and, in particular, avocado. This study investigated the ability of two calliphorids (*Calliphora dubia* and *Calliphora vicina*) and a syrphid (*Eristalis tenax*) fly species to pollinate Hass avocados in southwestern Australia. Four (4) field trials over three (3) years showed that each fly species (all found across Australia) was capable of pollinating Hass avocados when released into netted enclosures around multiple trees (12–26) during flowering. Trees enclosed with *Eristalis tenax* produced the highest fruit yield (18.0 kg/tree) outperforming trees pollinated by either *C. dubia* (11.6), managed honey bees in the open orchard (10.5) or *C. vicina* (6.8). Increasing fly numbers from 10,000 to 15,000 in the enclosures provided no additional pollination benefit. These results suggest that either *E. tenax* or *C. dubia* could be valuable managed pollinators for the avocado industry either with or without honey bees. *Calliphora dubia* was a significant pollinator during warmer flowering seasons and *C. vicina* was a useful pollinator during cold and wet flowering seasons.

## Simple Summary

Although flies regularly visit flowers, little research has gone into their pollination ability on commercial crops. An Australian project aimed to identify fly pollinators to secure insect pollinators into the future with a particular focus on avocados. This study investigated the ability of two calliphorids (*Calliphora dubia* and *Calliphora vicina*) and a syrphid (*Eristalis tenax*) fly (all found across Australia) to pollinate Hass avocados in south-western Australia. Three (3) years of field trial data show that all three species can pollinate Hass avocados when released into large enclosures around multiple trees during avocado flowering. Trees not enclosed (i.e., pollinated by bees and any wild insects in the orchard) produced the highest number of fruitlets/tree (89.8) followed by *E. tenax*-pollinated trees (79.8), *C. dubia* (43.4), honey bees only (38.2) and *C. vicina* (29.3). Pollination did not increase when using 15,000 flies compared to 10,000 flies, but almost twice as many fruitlets formed when using 10,000 flies (59) compared with 5000 flies (30.8) of either *C. dubia* or *C. vicina*. The one trial using *E. tenax* showed that it was a significant pollinator (18 kg/tree yield) whilst *C. dubia* was a good pollinator during warmer flowering seasons and *C. vicina* was a useful pollinator during cold and wet flowering seasons.

## 1. Introduction

Insect pollination is critical for the productivity of many food crops worldwide, with around 80% of these crops either depending on or benefiting from insect pollinators to enhance their yield [1]. Honey bees (*Apis mellifera* L. 1758) are the primary managed pollinator, servicing over 90% of these crops to meet their pollination needs [2]. However, global pollinator populations, including honey bees, have been declining steadily at an alarming rate in recent decades [3]. This is due to various stresses such as habitat fragmentation and loss [4], pesticide and herbicide residues [5], and climate change [3,6]. This decline and disappearance of honey bee species in the wild and the collapse of honey bee colonies has ecologists, beekeepers, and growers concerned about the sustainability of relying solely on honey bees for pollination [5].

To mitigate the risks associated with the decline of honey bees, it is essential to identify alternative or augmentative insect pollinators to support future pollination needs [7,8,9]. Despite the increasing demand for pollinator-dependent crops, the growth in the availability of managed honey bees has not kept pace [10]. As the second most important insects to visit flowers after honey bees, flies have shown potential in providing pollination services [2,11,12,13]. They are often active across a wider temporal range compared to honey bees [14,15], making them valuable for crops with variable floral receptivity [16,17]. Flies can be as efficient, or even superior, to honey bees in pollinating certain crops, including mango (*Mangifera indica* L.) [11,18,19], strawberry (*Fragaria* × *ananassa* Duchesne) [20], leek (*Allium ampeloprasum* L.) [21], onion (*Allium cepa* L.) [22], caraway (*Carum carvi* L.) [23], and oilseed rape (*Brassica napus* L.) [24]. Notably, several fly families, including Syrphidae (hover flies), Calliphoridae (blow flies) and Rhiniidae (snout-nosed flies), have been recorded in the literature as effective pollinators of various horticultural crops [9]. Flies visit the flowers of over 1000 plant species, with the diversity and frequency of these associations strongly suggesting that flies contribute significantly to pollination [25].

Syrphids and calliphorids are the two most significant non-bee pollinator taxa of pollinator-dependent crops globally [13]. Syrphidae (hover flies and flower flies) is one of the largest dipteran families with over 6000 species described worldwide [9]. In Cauca, Colombia, the syrphids (*Palpada scutellaris* Fabricius 1805 and *Ornidia obesa* Fabricius 1775) were regular visitors to avocado flowers [26]. Of the syrphid taxa reported to be visiting flowers in Australia, the introduced drone fly, *Eristalis tenax* L. 1758 has a worldwide distribution and high abundance throughout the year [27,28]. Syrphids obtain nearly all of their food resources as adults from flower nectar and pollen [29]. Under caged or greenhouse conditions, *Eristalis* spp. are efficient pollinators of crops such as onion and sweet pepper (*Capsicum annuum* L.), where they carry more pollen grains than nectar-collecting honey bees of equivalent size [30,31]. *Eristalis tenax* in particular has become popular within research on potential managed pollinators due to their abundance, behavioural plasticity, mobility and high fecundity, which are ideal traits for mass rearing and release [13,28,32,33]. *Eristalis tenax* is a proven pollinator of pak choi (*Brassica rapa* spp. *chinensis* L.) [34,35], kiwifruit (*Actinidia deliciosa* L.) [36], cranberry (*Vaccinium* subgenus *Oxycoccus* L.) [37], onion [28], sweet pepper (*Capsicum annuum* L.) [31] and carrot seed crops (*Daucus carota* L.) [15,38,39].

Calliphorids are important pollinators of crops such as mango in Thailand [40], Israel [19], Indonesia [41] and elsewhere [27] along with the pollination of seed crops in covered systems (e.g., bagged plants, small cages, tunnels and glasshouses) [9]. Seed yield of onions (*A. cepa*) was comparable to or better in caged plots using either *Calliphora vomitoria* L. 1758, *Lucilia ceasar* L. 1758 or *L. sericata* than yields from hand-pollinated or honey bee-pollinated plants [42,43]. Similarly, *C. vicina* is an effective pollinator of caged leek [15,21]. Calliphorid and sarcophagid flies are common, widespread pollinators of vegetable and forage seed crops, increasing the yield of brassica, carrot, pak choi, onion and radish; *Calliphora vicina* and *Pollenia* spp. (cluster flies) were the most abundant fly pollinators in Canterbury, New Zealand, during spring and summer [44].

Insects facilitate avocado (*Persea americana* Mill.) pollination, leading to increased fruit production, and there is evidence of yield improvements through improved pollination [45]. Most avocado producers use managed honey bees for pollination; however, honey bees can be sensitive to wind, rain and low temperatures [46,47] and often prefer other nectar sources to avocado [48]. This may reduce the contribution of honey bees to avocado pollination, hence a more diverse pollinator community including both wild and managed pollinators may provide more consistent pollination [49]. After honey bees, many wild insects contribute to avocado pollination including syrphids and calliphorids [45]. Like many insect-pollinated crops, avocado yields are at risk due to widespread pollinator declines [50,51]. Previous studies where avocado trees were denied access by insects resulted in close to zero fruit being set [45,52]. Studies carried out in Mexico showed that native species (e.g., stingless bees and flies) are as effective as the honey bee at avocado pollination [47,53,54]. The calliphorid fly *Chrysomya megacephala* Fabricius 1794 successfully pollinates avocados in Mexico [54] whilst in Colombia, a high diversity of calliphorids (*Lucilia eximia* Wiedemann 1819 and *Chrysomya putoria* Wiedemann 1830) were found visiting avocado flowers [26]. The pollination efficiency of the syrphid *Phytomia incisa* Wiedemann 1830 on avocado in Kenya was second behind *A. mellifera* with high pollen deposition rates and pollen grains on their bodies [55,56]. Similarly in avocados grown in Kandara, Kenya, the blow fly *Ch. putoria* and the syrphid *E. tenax* were the major flower visitors second behind *A. mellifera* [57].

Avocado production in Australia occurs over several growing regions including Queensland (Qld), New South Wales (NSW) and Western Australia (WA) to ensure a continuous supply year around [58], with Hass avocados being the dominant variety grown (≈80%) [59]. Sole reliance on honey bees for pollination needs is risky, especially given the predicted shortfall in honey bee hives needed to meet the growing pollination demands of the expanding avocado industry in WA as more avocado plantings come into production, which is currently 5283 ha [58]. This has intensified the need to identify alternative insects that can assist avocado pollination.

Flies from the Calliphoridae family have been observed visiting avocado flowers in Australia [60,61]; however, their pollination ability is only just being understood. In NSW, *C. vicina* was the dominant fly visiting avocado flowers (Finch, JTD Unpublished data). In the Tri-State region of Australia (where the borders of NSW, Victoria and South Australia meet), blow flies were the insect most frequently visiting avocado flowers, in addition to being the dominant pollinators of avocado [60]. The calliphorids recorded visiting avocado flowers (and potentially playing a significant role in their pollination) include *Calliphora stygia* Fabricius, *C. augur* Fabricius, *C. vicina* Robineau-Desvoidy, *Chrysomya rufifacies* Macquart, *Ch. varipes* Macquart, *Lucilia sericata* and *L. cuprina* Wiedemann [9,60,62]. *Calliphora dubia* and *C. albifrontalis* were first assessed in pollinating Hass avocados [61] based on observations of flower visitation by two closely related species, *C. augur* and *C. stygia*; these flies had >500 grains of avocado pollen on their bodies in orchards in Sydney, NSW [62,63]. Despite *C. albifrontalis* being an effective pollinator of glasshouse blueberries compared with no insect pollinator [64], this fly was not an effective pollinator of avocados [61], whereas *C. dubia* appeared to be a better pollinator of avocados compared with *C. albifrontalis* [61]. Both *C. albifrontalis* and *C. vicina* often feed on avocado flowers in WA [61].

Given that the syrphid *E. tenax* and the calliphorids *C. vicina* and *C. dubia* all occur predominantly in the southern half of Australia, where the avocado industry is rapidly expanding, they are likely candidates for use as managed pollinators to support honey bee pollination of avocado. The goal of this study was to assess the pollination efficiency and contribution to fruit yield in avocado trees by these three fly species in south-western Australia. It builds on the investigations by Cook et al. (2023) [61] and used field approaches, where multiple avocado trees were enclosed in netting. The findings will provide insight into the potential use of these fly species as managed pollinators in avocado production.

## 2. Materials and Methods

### 2.1. Fly Colonies for Use in Multi-Tree Enclosure Trials

**Figure 1:**
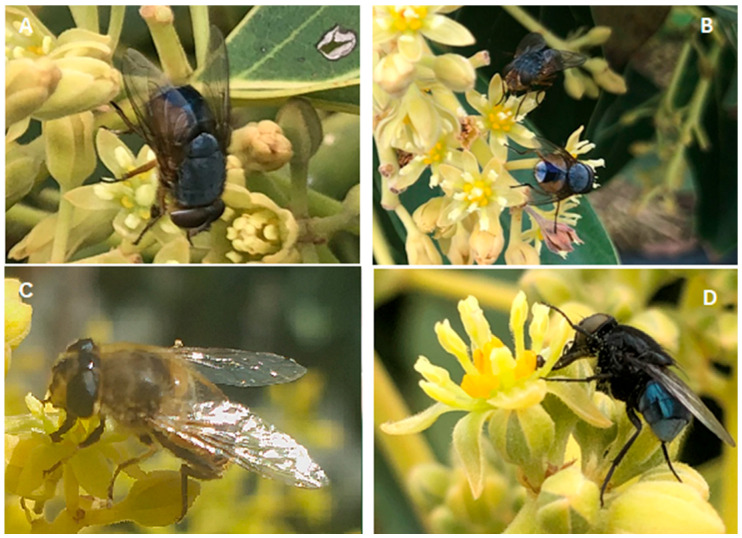
The fly species tested for their ability to pollinate Hass avocados as a result of feeding on their flowers: the western, blue-bodied blow fly *Calliphora dubia* (**A**,**B**), the drone fly *Eristalis tenax* (**C**) and the European blue bottle blow fly *Calliphora vicina* (**D**).

Colonies of the two species of calliphorid were set up from field-caught adult flies. *Calliphora dubia* (Figure 1A,B) adults (minimum of 50) were collected from protein-baited fly traps (baited with 250 g of beef liver and 125 mL of a 1.5% sodium sulphide solution) (Solar Fly Traps^®^ from Arbico Organics (Tucson, AZ, USA) set out in bushland at Yanchep (50 km north of Perth; −31.832049° S, 115.837055° E), Toodyay (85 km northeast of Perth; −31.3300° S, 116.2801° E), Lancelin (85 km north of Perth; −31.0119° S, 115.2010° E) and Bullsbrook (40 km northeast of Perth; −31.7323° S and 115.9906° E)). Similarly, *C. vicina* (Figure 1D) adults were collected from the same fly traps placed at several locations in (a) the south-west of Western Australia (Preston Beach (−32.91854° S, 115.71296° E), Capel (−33.52121° S, 115.56024° E) and Busselton (−33.64165° S, 115.46172° E)); and (b) Tasmania (Bellerive (−42.87905° S, 147.39662° E), South Hobart (−42.89213° S, 147.29803° E), Sandy Bay (−42.89938° S, 147.32611° E) and Kellevie (−42.78305° S, 147.79827° E)). Each protein-baited fly trap had water and sugar inside the catch section of the trap, so that the adult flies caught would survive for 3–4 days before being brought back to the laboratory. Each entire fly trap was placed in a 4 °C room to chill and then sort the adult flies caught to remove *C. dubia* and *C. vicina* adults and place into separate cages (60 cm × 60 cm × 60 cm). Cages of the field-collected adults (60 cm^3^) were supplied with water, a 50:50 mixture of sugar and milk powder, and were held at 25 °C ± 1 °C and a photoperiod of 14:10 h (L:D).

Beef liver cut into small cubes was used to protein feed the adults to elicit the laying of live larvae (in the case of *C. dubia*) and eggs (from *C. vicina*). First instar larvae of each blow fly species were then placed onto a rearing medium containing 90% meatmeal and 10% whole dried egg powder (*v*/*v*). The dry ingredients of meat meal (Talloman Rendering, Hazelmere, Perth, WA, Australia) and whole dried egg powder (Farm Pride Foods, Keysborough, VIC, Australia) were mixed first before adding water to produce a moist larval substrate. Both species of fly were in laboratory culture for ten generations before being used in this study, where newly emerged adults were released into large net enclosures. Approximately 5000 pupae of *E. tenax* were sourced from SeedPurity Pty Ltd. (Margate, TAS, Australia) who have a small-scale rearing population for research purposes. Adult *E. tenax* were allowed to emerge in a large 1 m^3^ cage for 2 days prior to transportation to Capel Farms in WA and release into the netted enclosure around avocado trees (Figure 1C).

### 2.2. Multi-Tree Enclosures

Trees designated as “open” pollinated controls were pollinated by managed honey bees brought onto the orchard at the start of flowering and any other wild insect pollinators in the orchard. With each field trial, a number of insect-proof enclosures were erected around avocado trees in commercial orchards prior to the commencement of flowering. The netting material used for each multi-tree enclosure was Vege netting (light netting 45 g/m^2^ with >8000 threads/cm^2^) from Commercial Netmakers, Bibra Lake, Perth, WA. The enclosures covered either one, two or three rows of trees (14–39 trees) including Hass and Type B cultivars (Edranol or Ettinger). The only source of sugar for flies in the enclosures was the avocado flowers and tree irrigation provided adequate water for the flies within the enclosures. The same number of trees outside and near the enclosures was marked as open pollination for this and all subsequent trials. In some trials, a small nuc hive of bees was placed inside an enclosure to determine pollination by bees only.

#### 2.2.1. Busselton (2021)

**Figure 2:**
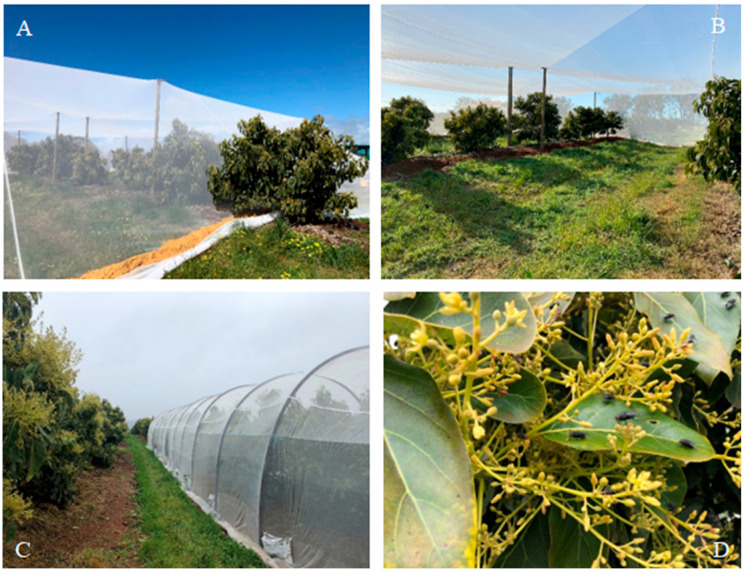
Fly-proof enclosures built around avocado trees in the south-west of WA that covered either three rows of avocado trees at Busselton (**A**), two rows of trees at Capel (**B**) or a single row of avocado trees at Pemberton (**C**). Newly released flies in an enclosure resting on avocado leaves (**D**).

At Ruabon Farm in Ludlow, WA (−33.624555° S, 115.473535° E), 12 km north of Busselton, four (4) separate fly-proof enclosures were constructed with each covering three rows of avocado trees (36 Hass and 3 Ettinger) (Figure 2), with thirteen avocado trees/row (see Figure 2A). A small nuc hive of honey bees, equivalent to 3.5 hives/ha, was introduced into one of the enclosures.

#### 2.2.2. Pemberton (2021)

At Delroy Orchards in Channybearup, 10 km northwest of Pemberton, WA (−34.373028° S, 115.968435° E), three separate enclosures covered 14 trees along the same row (12 Hass and 2 Edranol). A small nuc hive of honey bees, equivalent to 3.5 hives/ha, was introduced into one of the enclosures.

#### 2.2.3. Capel (2022)

Four (4) large fly-proof enclosures were erected at Capel Farms in Capel (−33.5153822° S, 115.5603244° E), 200 km south of Perth, with each enclosure covering two rows of avocado trees (24 Hass and 3 Ettinger).

#### 2.2.4. Capel (2023)

Three large enclosures were erected at Capel Farms in late September, 2023. Each enclosure covered two rows of avocado trees (26 Hass and 3 Ettinger).

### 2.3. Fly Releases into the Enclosures

One week prior to releasing adult flies into the enclosures, the inside of each enclosure was sprayed with Success^®^ to kill any flying insects (bees, flies, beetles wasps) trapped inside the mesh during construction. The mean numbers of each fly species released into the enclosures across the field trials are shown in Table 1. The number of flies released into the enclosures was based on the number of honey bees used in commercial avocado production with the aim of having a similar number of flies/tree as there were honey bees/tree. In the orchard trial sites, 2.5–3.5 hives/ha of honey bees were introduced, which translates to 112,500–157,500 bees/ha. The orchard tree density of 320 trees/ha means that ≈350–500 honey bees are available/tree. Since only 25–33% of honey bees in a hive forage at a given time [65,66], we assumed that between 100 and 145 honey bees/tree were out foraging in the orchard [61], which meant releasing similar numbers of flies/tree in each enclosure.

Flies were introduced into the enclosures the day after managed honey bee hives were brought into the orchard. The number of flies within each enclosure was calculated by the number released and factoring in a 33% decline in survival each week thereafter as we assumed that flies released into the enclosures would live ≈ 3 weeks [61]. Subsequent releases of adult flies were made every 2 weeks after the first release (60% of initial release number) to account for adult mortality and to keep the adult fly numbers consistent. It is not easy to put an exact number of flies into an enclosure as it is difficult to predict the % adult emergence from a given cohort of pupae. One week after all adult flies were released into each enclosure, a count was made of the number of pupal exuviae from each cage to determine the exact number of adult flies that emerged. All fly enclosures were dismantled once all bee hives had been removed from the orchard at the end of flowering.

### 2.4. Methods of Assessment and Data Collection

Fruitlet counts were performed 6–7 weeks after flowering had ended on every individual Hass tree within each enclosure as well as the same number of Hass trees nearby in the open orchard. Type B trees (Edranol or Ettinger) were not assessed for fruitlet or mature fruit production. Counts were made only of fruitlets ≥5 mm in diameter, which, according to Sedgley (1980) [67], means that the fruit are normal and 100% fertilised; these fruitlets form within 3 weeks after flowering has ended. A count of mature fruit harvested (number and weight) was conducted in June-July of the following year as the time from flowering to harvest takes ≈8–9 months.

#### Visual Scoring System to Estimate Fruit Yield

In the 2023 field trial, a novel visual scoring system was tested to evaluate individual trees based on the number of fruitlets per tree across all treatments 3 weeks after flowering had ended. Trees were scored on a scale from 1 (lowest fruitlets/tree) to 5 (highest fruitlets/tree). Mean visual scores for each treatment were calculated by scoring each half of the tree and then averaging the scores across all Hass avocado trees within each enclosure. This was compared with the actual fruitlet counts performed on the same trees 8 weeks after flowering had ended.

### 2.5. Weather Data

Weather data for each trial site and year were extracted from the following sources: Busselton data was extracted from the nearest Bureau of Meteorology weather station, Busselton Aero (Site number 009603), which is 7 km south-west of the trial site. Pemberton data was extracted from the DPIRD weather station Channybearup (CL001), which was 8 km south-west of the trial site; Capel data was extracted from an on-farm weather station at Yalyalup.

### 2.6. Statistical Analysis

Avocado yield attributes were analyzed in R (version 4.1.1) R Core Team (2021) [68] using mixed-effects modelling to account for the experimental design structure. 

Count data (e.g., fruitlets and mature fruits) was modelled using generalized linear mixed models (GLMMs) with a negative binomial distribution via the “*glmmTMB*” package. Fixed effects included pollinator species (treatment), pollinator density, site, and year, along with their interactions. Random intercepts were specified for enclosure nested within site and year to account for the non-independence of trees within enclosures. The model formula was structured as: response ~ treatment * density + (1 | site:year) + (1 | enclosure). This specification treated the enclosure as the experimental unit, thereby avoiding pseudo-replication due to clustering of multiple trees within enclosures. Post-hoc pairwise comparisons of estimated marginal means (EMMs) were performed by using the “*emmeans*” package with multiplicity-adjusted contrasts by employing the Benjamini-Hochberg correction to control the false discovery rate (FDR), ensuring robust inference while maintaining statistical power across multiple comparisons.

For continuous data (e.g., fruit weight or yield–kg/tree), linear mixed-effects models (LMMs) with Gaussian distribution were fitted using the “*lme4*” package, employing the same fixed effects structure and random intercepts for site-by-year and enclosure using this formula: yield ~ treatment * density + (1 | site:year) + (1 | enclosure). Tukey’s Honest Significant Difference (HSD) test was applied to identify statistically significant differences at the 5% significance level.

Model selection was guided by the Akaike Information Criterion (AIC), with preference given to models with lower AIC values. Model diagnostics checks included examination of residual plots to verify assumptions of normality and homogeneity of variance for the LMMs. For GLMMs evaluation, dispersion parameters were checked to confirm appropriate model fit.

## 3. Results

### 3.1. Year 1 Field Trial, Busselton (2021)

Weather conditions during the 2021 flowering period (43 days) were particularly cold and wet (see Table 2). The average maximum and minimum temperatures were both lower (by >1 °C) than the long-term average (LTA), and the rainfall and number of rain days were both ≈ three times the LTA. These persistent conditions during flowering (Table 2) significantly reduced *C. dubia* emergence, resulting in an average of only 2754 flies in the enclosure over the flowering period (i.e., 76 flies/tree). When expressed per 5000 flies, *C. dubia* achieved similar pollination to that in the open pollination treatment (*p* > 0.05).

**Figure 3:**
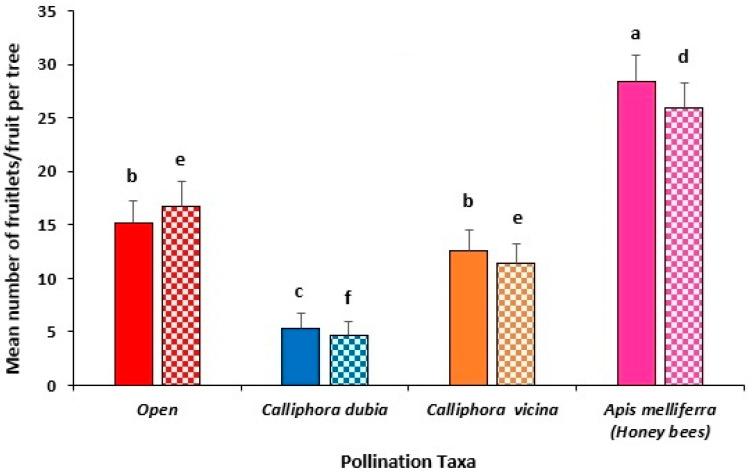
Mean number of avocado fruitlets (solid fill) and mature fruit at harvest (pattern fill) of Hass trees in multi-tree enclosures during 2021 flowering season at Busselton, southwest of WA. Enclosed trees were either pollinated by 10,000 *Calliphora dubia* (blue), 5000 *Calliphora vicina* (orange) or a small nuc hive of honey bees (pink) and compared with trees in the open orchard (red) near to the enclosures (designated as Open) pollinated by managed honey bee hives in the orchard. Standard error bars are indicated and different letters indicate significant differences between treatments (*p* ≤ 0.05, Tukey’s HSD test) when comparing fruitlet counts (a–c) or mature fruit counts (d–f) separately.

Trees enclosed with a small honey bee hive produced a significantly (*p* < 0.001) higher number of fruitlets (31.9 ± 4.7), compared to *C. vicina* (13.4 ± 3.3), open-pollinated trees (12.5 ± 3.3) and *C. dubia* (4.2 ± 1.1), which were not significantly different (*p* > 0.05) (Figure 3).

There was no significant difference in the number of mature fruit at harvest between the honey bee enclosure (22.3 ± 3.0 fruit) and open-pollinated trees (14.4 ± 3.3 fruit) (*p* > 0.05). *Calliphora vicina*-pollinated trees produced significantly less fruit (10.2 ± 2.4 fruit) than those pollinated by honey bees only (*p* < 0.05), while *C. dubia* produced the lowest number of fruit per tree (3.5 ± 0.7 fruit), significantly less (*p* < 0.05) than all other pollination taxa (Figure 3).

Total fruit yield/tree was not significantly different between honey bees-only (4.8 ± 0.6 kg/tree) and open-pollinated trees (3.6 ± 0.8 kg) (*p* > 0.05) but both treatments had a significantly higher fruit yield than *C. dubia* pollinated trees (0.9 ± 0.2 kg/tree) (*p* < 0.05). Although there was no significant difference in fruit yield between *C. vicina*-pollinated trees (2.4 ± 0.5 kg) and *C. dubia*-pollinated trees, trees enclosed with honey bees only produced significantly more fruit than those enclosed with either *C. vicina* or *C. dubia* (*p* < 0.001).

### 3.2. Year 1 Field Trial, Pemberton (2021)

The average maximum and minimum temperatures during the flowering period (42 days) were very similar to the LTA’s as was the monthly rainfall and number of rain days (Table 2).

**Figure 4:**
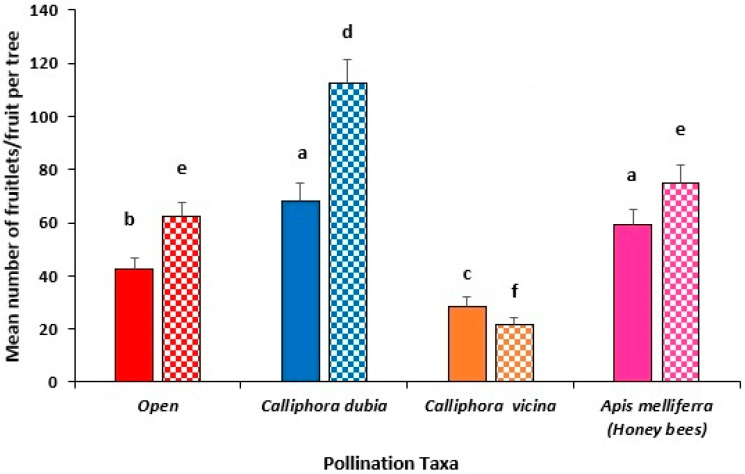
Mean number of avocado fruitlets (solid fill) and mature fruit at harvest (pattern fill) of Hass trees within multi-tree enclosures during the 2021 flowering season at Pemberton in the southwest of WA. Enclosed trees were either pollinated by 5000 *Calliphora dubia* (blue), 5000 *C. vicina* (orange) or a small nuc hive of honey bees (pink) and were compared with trees in the open orchard (red) near to the enclosures (designated as Open) pollinated by managed honey bee hives in the orchard. Standard error bars are indicated and different letters indicate significant differences between treatments (*p* ≤ 0.05, Tukey’s HSD test) when comparing fruitlet counts (a–c) or mature fruit counts (d–f) separately.

The number of fruitlets 6 weeks after flowering were not significantly different between any of the insect pollination taxa (*p* > 0.05) (Figure 4). In contrast to the Busselton site, *C. dubia* produced 40% more fruitlets/tree (79.9 ± 20.1) than honey bees only (57.2 ± 15.3) and 82% more than open-pollinated trees (43.8 ± 8.2), while *C. vicina* produced the lowest number of fruitlets/tree (30.1 ± 6.1).

Although *C. dubia*-pollinated trees had the highest number of mature fruits (91.9 ± 24), it was not significantly more than either honey bees only (62.9 ± 17.8) or open pollination (63.4 ± 10.9), respectively (*p* > 0.05), but was significantly more than *C. vicina*-pollinated trees (20.5 ± 4.9) (*p* < 0.01) (Figure 4). The number of avocado fruit for *C. dubia*, open pollination and honey bees only treatments were statistically similar (*p* > 0.05).

A similar trend was recorded in fruit yield per tree, where *C. dubia*-pollinated trees had the highest fruit yield (23.7 ± 4.8 kg/tree), significantly (*p* < 0.01) more than *C. vicina* (4.8 ± 1.2 kg) but not significantly more than either open-pollinated trees (17.9 ± 3 kg) or those in an enclosure with honey bees (14.4 ± 3.9 kg) (*p* > 0.05).

### 3.3. Year 2 Field Trial, Capel (2022)

The average maximum and minimum temperatures were both lower (by >2 °C) than the LTA’s during the flowering period (56 days). When each enclosure was checked one week after the first fly releases, it was apparent that the emergence of *C. dubia* adults was severely compromised due to the very cold nights and high humidity (daily min temp of less than 3 °C and humidity of 99%), which affected the opening and drying of their wings. Due to the significant loss of *C. dubia* adults, it was decided to abandon this treatment.

**Figure 5:**
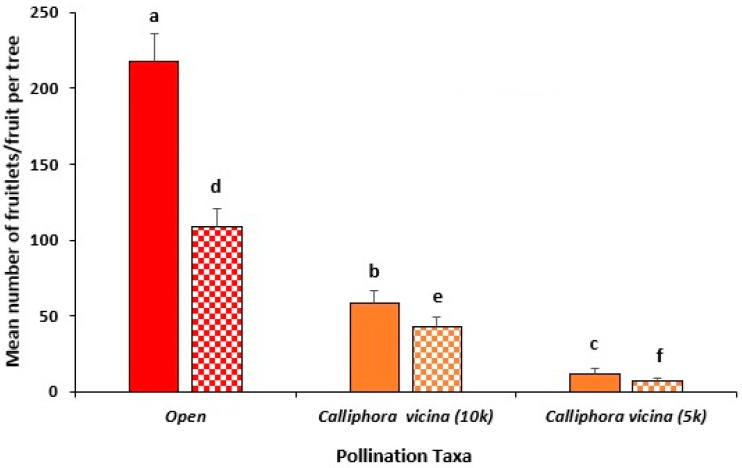
Mean number of avocado fruitlets (solid fill) and mature fruit at harvest (pattern fill) of Hass trees in multi-tree enclosures during the 2022 flowering season at Capel in the south-west of WA. Enclosed trees were either pollinated by 5000 or 10,000 *Calliphora vicina* flies (orange) and were compared with trees in the open orchard (red) pollinated by managed honey bee hives (designated as Open). Standard error bars are indicated and different letters indicate significant differences between treatments (*p* ≤ 0.05, Tukey’s HSD test) when comparing fruitlet counts (a–c) or mature fruit counts (d–f) separately.

Open-pollinated trees produced the highest number of avocado fruitlets (248.8 ± 22.9), significantly (*p* < 0.001) exceeding *C. vicina* (64.4 ± 12.6) (Figure 5). A higher number of *C. vicina* (10,000 vs. 5000) resulted in significantly more fruitlets/tree (64 vs. 12; *p* < 0.05).

Similarly, open-pollinated trees yielded significantly (*p* < 0.01) more mature fruit per tree (113.4 ± 11.6) compared to *C. vicina* (69.2 ± 10.2) (Figure 5). Among *C. vicina* fly treatments, 10,000 adult flies led to significantly more mature fruit at harvest/tree than 5000 adult flies (69 vs. 9; *p* < 0.01) (Figure 5).

Both *C. vicina* with a 10,000 fly density and the open pollination treatments produced comparable (*p* > 0.05) yields of mature fruit at harvest (22 and 19 kg/tree, respectively). Conversely, the lower fly numbers of *C. vicina* (i.e., 5000) resulted in a significantly lower fruit yield (12 kg/tree) (*p* < 0.05).

### 3.4. Year 3 Field Trial, Capel (2023)

In contrast to 2022, the 2023 flowering period was very dry, with maximum temperatures ≈ 2 °C warmer in October and temperatures during November being similar to LTAs (Table 2).

Pollination performance varied significantly across treatments, with *E. tenax* producing the highest number of fruitlets (79.8 ± 11.8), which was significantly more than both open-pollinated trees (41.9 ± 6.2) (*p* < 0.05) and trees pollinated by *C. vicina* (29.6 ± 4.8; *p* < 0.01); however, there was no significant difference between *E. tenax* and *C. dubia* (66.1 ± 11.6) fruitlet numbers (*p* > 0.05).

A similar trend was observed in the mature fruit at harvest, with *E. tenax* producing more mature fruit (73 ± 9.4) compared with both open pollination (44.8 ± 7.9) (*p* < 0.05) and *C. vicina*-pollinated trees (27.4 ± 4.3) (*p* < 0.01), but not significantly more than *C. dubia*-pollinated trees (57 ± 10.5) (*p* > 0.05).

**Figure 6:**
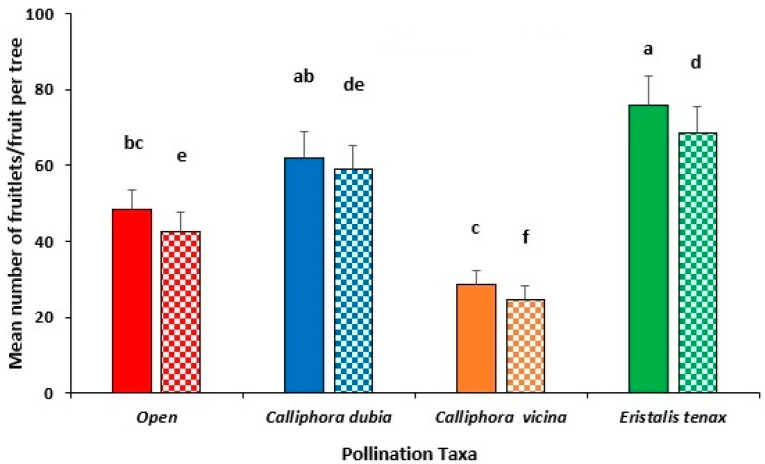
Mean number of avocado fruitlets (solid fill) and mature fruit at harvest (pattern fill) of Hass trees in multi-tree enclosures during the 2023 flowering season at Capel in the south-west of WA. Enclosed trees were either pollinated by *E. tenax* (green), *C. dubia* (blue) or *C. vicina* (orange) flies and were compared with trees in the open orchard (red) pollinated by managed honey bee hives. Standard error bars are indicated and different letters indicate significant differences between treatments (*p* ≤ 0.05, Tukey’s HSD test).

This trend was similar to the avocado fruit yield/tree (Figure 6) where trees pollinated by *E. tenax* produced the highest fruit yield (18.4 ± 2.3 kg/tree), which was not significantly more than either *C. dubia* (14.7 ± 2.4 kg) or open-pollinated trees (11.5 ± 1.8 kg) (*p* > 0.05); however, all treatments had a higher fruit yield/tree than *C. vicina*-pollinated trees (7.8 ± 1.3 kg) (*p* < 0.01).

#### Testing of Visual Scoring System to Estimate Fruit Yield

**Figure 7:**
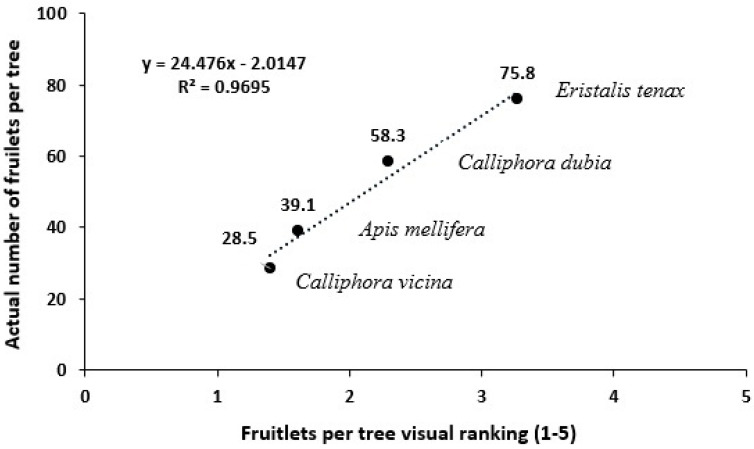
Correlation between mean visual ranking score and mean actual fruitlet count per tree of Hass avocado trees from the 2023 Capel field trial. Means are indicated for fly taxa within each enclosure of *E. tenax*, *C. dubia*, *C. vicina* and *A. mellifera* (honey bees).

There was a strong positive relationship between the visual ranking of fruitlets per tree and the actual fruitlet count of avocado trees within each enclosure across all treatments (Figure 7). The trendline equation (y = 24.476x − 2.0147) demonstrated that the visual ranking scale (1 to 5) is an effective predictor of the actual fruitlet count with a high coefficient of determination (R^2^ = 0.9695) indicating a 97% accuracy in the relationship. The treatments exhibited significant differences in fruitlet production. For example, *E. tenax* resulted in the highest fruitlet count per tree (visual ranking score of 5; corresponding to 75.8 actual fruitlets), while *C. vicina* produced the lowest fruitlet count (visual ranking score of 1; with 28.5 actual fruitlets).

### 3.5. Comparative Analysis Across All Field Trials

Statistical analysis confirmed significant effects (*p* < 0.001) of treatment type (honey bees, each fly species and open pollination), fly density (5, 10 and 15 thousand), trial site (Capel, Pemberton or Busselton), and year, with a notable interaction between treatment x fly density (*p* < 0.05). No significant difference was observed between releasing 10,000 (58.7) or 15,000 (49.0) adult flies per enclosure (*p* > 0.05), although both fly densities outperformed the 5000-fly treatments (28.0). The interaction between fly density x site was not significant (*F* = 1.711, *df* = 2, *p* > 0.05).

Site-wise comparison revealed that fruitlet production per tree was highest at Capel (78.1), followed by Pemberton (51.6) and Busselton (15.5) (*p* < 0.05). Across the three-year trials (Figure 7), open-pollinated avocado trees (pollinated by honey bees and wild insects) produced significantly more fruitlets per tree (89.8) than trees pollinated by *E. tenax* (79.8; *p* < 0.05). In turn, trees enclosed with *E. tenax* outperformed *C. dubia* (43.4; *p* < 0.05). Both fly species significantly outperformed honey bees only (38.2) and *C. vicina* (29.3) (*p* < 0.05). Seasonal variation was substantial (*p* < 0.001), with mean fruitlet production peaking in 2022 (110), followed by the 2023 (55) and 2021 (24) growing seasons.

In contrast to the fruitlet data, results for avocado fruit yield by weight (kg/tree) showed that *E. tenax*-pollinated trees yielded significantly more mature fruits (73.0) than open-pollinated trees (56.8; *p* < 0.05). Open-pollinated trees also outperformed *C. dubia*-pollinated trees (48.5), which in turn produced more mature fruits than those in honey bees-only (32.6) and *C. vicina* (27.8) enclosures (*p* < 0.05).

Avocado fruit yield (kg/tree) data showed trends across treatments, fly densities, and years, though differences were not statistically significant (*p* > 0.05). *Eristalis tenax* pollinated trees had the highest average fruit yield (17.42 kg/tree), followed by *C. dubia* (14.8 kg), open-pollinated trees (10.4 kg), and *C. vicina* (6.8 kg). Avocado yields were highest in 2022 (18.3 kg/tree), representing a 37% increase over 2023 (13.3 kg/tree). Furthermore, the 10,000-fly density treatment produced the highest yield (19.1 kg/tree), outperforming both the 5000 fly density (17.7 kg/tree) and 15,000 fly density (11.5kg/tree) treatments.

## 4. Discussion

Despite flies being regular visitors to flowers, little research has been conducted into their pollination ability, in particular, on commercial fruit crops [13]. A national project in Australia aimed to identify potential fly pollinators to secure additional pollination services into the future for the Australian horticulture industry with a particular focus on the avocado industry. This study further investigated the pollination potential of two calliphorids (*C.dubia* and *C. vicina*) and a syrphid (*E. tenax*) fly species on Hass avocados in south-western Australia. Four (4) field trials over three (3) years show that all three fly species are capable of pollinating Hass avocados when released into large insect-proof enclosures around multiple trees during avocado flowering. The enclosures used in these trials provided a more open environment for flies to forage among flowers compared with the paired-tree enclosures used in the study by Cook et al. (2023) [61]. This provided a more realistic assessment of the pollination ability of the three fly species used in this study. Both fruitlet formation (6 weeks after flowering) and mature fruit at harvest (8–10 months after flowering) were used to measure pollination success and fruit set. The multi-tree enclosures ≥ two Type B avocado varieties (Edranol and Ettinger) with Hass avocados (Type A) as fruit production is best when Type A and Type B varieties are interplanted [48,69].

During the first year of field trials in 2021, persistent cold and rainy periods during the month of October significantly affected the emergence of *C. dubia* adults at Busselton when the unhatched pupae were left in the enclosures, resulting in the number of *C. dubia* adults in the enclosure being only 2754 (i.e., 76 flies/tree). This was in contrast to *C. vicina,* where pupal emergence was >95% and 9444 flies or 255 flies/tree were in the enclosure. When correcting for the lower number of *C. dubia* in the enclosure so that they were equivalent to the number of *C. vicina*, their pollination success was slightly more than open-pollinated trees. Greater pollination within the honey bee enclosure compared to both fly and open pollination treatments was likely due to the thermal effect of the fine insect-proof mesh, which would restrict air flow and raise internal temperatures [70,71]; this would promote a wider time-period of honey bee foraging relative to managed honey bees in the open orchard where conditions would have been colder. Within the enclosure, honey bees were limited to the trees available for their foraging needs, with no issue of competing bloom (as opposed to honey bees foraging in the open pollination treatment). Further, the shorter foraging distances from the hive within the enclosure compared to the position of the hives servicing the open pollination treatment may have contributed to the higher pollination observed. Different bee species prefer to forage at different temperatures [72] and the foraging activity of *A. mellifera* is low at ambient temperatures below 12–14 °C and solar radiation under 500 lux [2,73,74,75], conditions which frequently occur in early to mid-spring in the south-west of WA. Based on the avocado farm manager’s definition of a pollination event occurring (3 nights equal to or above 10 °C followed by 3 days equal to or above 17 °C, where there is the highest probability of male and female flowers being opened at the same time), there were only 5 “pollination events” at Ruabon Farm (Busselton) in the 2021 flowering season compared with 10 in 2018 and 12 in the 2019 flowering season.

The two species of calliphorid flies selected to first assess the pollination of Hass avocados [62] was based on observations of flower visitation by two closely related species, *Calliphora augur* and *Calliphora stygia*; these flies had >500 grains of avocado pollen on their bodies in orchards in Sydney, NSW [63]. In WA, *C. dubia* is the sister species to *C. augur*, whilst *C. albifrontalis* is the sister species to *C. stygia* [76]. Despite *C. albifrontalis* being an effective pollinator of glasshouse blueberries when present during flowering compared with bushes without any insect pollinator [64], this fly was not an effective pollinator of avocados [61]. *Calliphora dubia* appeared to be a better pollinator of avocados in paired-tree enclosures relative to *C. albifrontalis* (despite their not being a control treatment where no insects could access enclosed avocado trees) [61]. Both *C. albifrontalis* and *C. vicina* often feed on avocado flowers in WA [61], whereas in NSW, *C. vicina* was the dominant fly visiting avocado flowers (Finch, JTD Unpublished data).

Successful pollination in this study was measured by the formation of fruitlets (>5 mm in diameter) [67] along with the fruit yield at harvest. In each year’s crop production, there was a fruit drop event prior to final harvest; this often occurs in avocado trees, coinciding with the summer growth flush, and can be a major limiting factor in avocado production during a season of heavy fruit load [77]. Excessive fruit abscission often contributes to alternate bearing, i.e., the production of a heavy crop yield being followed by a light crop yield and is characteristic of many avocado cultivars [78]. This was observed in this study, where the 2022 season produced more fruitlets and subsequently more avocado fruit compared to other seasons (2021 and 2023). Excess flower production provides the opportunity for selective fruit drop early in their development. Fruits derived from outcrossing show higher chances of reaching maturity [79,80]. Estimating avocado yield soon after flowering is often challenging and labour-intensive. A novel, visual ranking system was tested in this study to address this challenge and proved to be highly effective. With >95% accuracy, this approach offers a reliable and efficient tool for predicting avocado fruit production, reducing reliance on time-consuming counting of fruitlets for yield data. Furthermore, the outcomes of the visual ranking system aligned closely with the final fruit harvest data, reinforcing the validity and efficacy of this method as a practical tool for yield estimation.

Most avocado orchards in Australia use managed honey bee hives to improve crop yield. This study has been the second in a series of field-based enclosure trials showing that flies can pollinate avocado flowers as a result of their feeding on the flowers to obtain nectar. Both calliphorid fly species tested are native to Australia and are present in southwest WA during September through to November when the trials were conducted. *Calliphora dubia* is endemic to mainland Australia except for the eastern coastline, where its sister species, *C. augur* (the lesser brown blowfly), occurs [81]. Cook et al. (2020) [9] highlighted calliphorid species that have been recorded visiting avocado flowers and/or playing a role in their pollination in Australia, which include the introduced species *C. vicina*, which is now widespread across the southern half of Australia and is found worldwide. Much is already known about this fly in the context of forensic entomology, where developmental rates of the pre-adult stages (eggs, larvae, pupae) have already been reported [82,83,84] and methods to mass rear this species are well documented [85]. The blow fly species tested are large and hairy and regularly visit flowers for nectar [86]. Avocado flowers within the enclosures provide nectar to support flight and in the case of the cosmopolitan green blowfly, *L. sericata*, ingested pollen from feeding on Oxeye daisy (*Leucanthemum vulgare* Lam) helps to support oocyte development [87].

Reports that blow flies discriminate less between crop lines than honey bees [88,89] and that *E. tenax* similarly forages randomly among vegetable seed lines [90] together suggest that these flies would be effective in cross-pollinating Type A and Type B avocado varieties. Hass avocado trees selectively retain cross-pollinated fruitlets, which are larger than self-pollinated fruitlets and ultimately produce larger fruit [80]. There was no significant difference in fruit production between 10,000 and 15,000 adult fly densities, suggesting floral resource limitation with more flies in the enclosures. This cross-site and multi-year analysis reinforces the potential of *E. tenax* and *C. dubia* as effective managed pollinators in avocado production systems. The findings support the hypothesis that some fly taxa can supplement or even rival traditional honey bee pollination under orchard conditions. For example, the mean fruit yield/tree (kg) when enclosed with either *E. tenax* flies (17.4) or *C. dubia* flies (14.2) in the Capel 2023 trial was 67 and 36% higher, respectively, compared with open-pollinated trees (10.4) and trees enclosed with *C. vicina* flies (6.8 kg). Relative to honey bee only pollinated trees, *E. tenax* increased yield by 96%, *C. dubia* by 69% and open pollination increased it by 46%, whilst *C. vicina* yielded 13% less.

Despite four field trials being conducted, there was considerable variation in weather conditions across flowering seasons, which indicates the crucial role this plays in avocado pollination success and fruit development. This altered the number of “pollination events” during flowering as well as the performance of each insect taxa. The series of field trials in this study confirmed that *E. tenax* was an efficient pollinator of avocados in multi-tree enclosures with Hass and Type B varieties, whilst *C. dubia* was a significant pollinator during warmer flowering seasons and *C. vicina* was a useful pollinator during cold and wet flowering seasons. Trees enclosed with *C. vicina* had one-third less fruit compared to open trees pollinated by bees, which may have been due to the warmer and drier flowering season as *C. vicina* is predominantly found in the southern half of Australia [61,81] and is a cool-adapted fly species [91]. This study also identified that newly emerged adult fly releases are preferred over leaving fly pupae in an orchard, especially if very cold and/or humid nights are expected, which significantly impact adult fly emergence.

Global pollinator decline [92] is likely to adversely impact avocado yields as demand for avocado continues to rise, as production has risen from 2.7 million tonnes in 2000 to 10.5 million tonnes in 2023 [93]. Increased avocado plantings has resulted in adverse environmental impacts such as biodiversity decline in some growing regions [94]. Avocado yields have increased in Chile, where either native vegetation has been maintained around orchards or strips of flowering plants have been established throughout the orchard. The resultant increase in fruit production was most likely due to increased flower visits by flies and other wild insects [95]. Modifying the understory and vegetation between tree rows could provide habitat to support flies and other wild pollinators to enhance pollinator abundance and diversity around orchard crops [96,97,98,99,100] including avocado orchards [101]. For example, more insects were caught in Australian almond orchards with diverse ground cover than in orchards without ground cover, with tachinids and blow flies being the most abundant fly families [102].

## Figures and Tables

**Table 1:** Numbers of insect pollinator species within each multi-tree enclosure by site and year in avocado orchards in the south-west of Western Australia. The expected and actual (italicised) numbers of adult flies are given for each enclosure. Avocado production from each enclosure was compared with open-pollinated trees (i.e., control; honey bees and other insects).

Site	Year	Insect Pollinator Species Within Enclosures					
		*Apis mellifera*	*Calliphora vicina*		*Calliphora dubia*		*Eristalis tenax*
Busselton	2021	5k	5k (*3046*)	*-*	5k (*2754*)		*-*
Pemberton	2021	5k	5k (*3244*)	-	5k (*2815*)	-	-
Capel	2022	-	5k (*3899*)	10k (*7899*)	-	-	-
Capel	2023	-		10k (*9783*)		10k (*11,050*)	5k (*3532*)

5k = 5000; 10k = 10,000 and 15k = 15,000 adult flies released into the enclosures.

**Table 2:** Weather conditions during the flowering period (FP) at each avocado orchard in southwestern WA during each year (2021–2023) and compared with the long-term local averages (LTA’s) *.

Year	Site	Month	Max (°C)		Min (°C)		Rainfall (mm)		Rain Days	
			FP	LTA	FP	LTA	FP	LTA	FP	LTA
2021	Busselton (a) *	Oct	**19.8**	21.4 ^1^	**6.4**	8.7 ^1^	109	32 ^1^	16	6 ^1^
		Nov	**22.6**	25.1 ^1^	**8.2**	10.7 ^1^	2	22 ^1^	2	4 ^1^
2021	Pemberton (b)	Oct	19.0	18.8 ^2^	8.6	8.6 ^2^	98	93 ^2^	11	12 ^2^
		Nov	**19.2**	21.4 ^2^	**8.5**	10.3 ^2^	17	48 ^2^	9	8 ^2^
2022	Capel (c)	Oct	**18.8 ^3^**	21.4 ^4^	**7.0 ^3^**	8.7 ^4^	35	42 ^4^	11	9 ^4^
		Nov	**21.6 ^3^**	25.1 ^4^	**8.8 ^3^**	10.7 ^4^	16	18 ^4^	6	4 ^4^
2023	Capel (c)	Oct	**23.3**	21.4 ^4^	8.9	8.7 ^4^	9	42 ^4^	3	9 ^4^
		Nov	24.8	25.1 ^4^	10.2	10.7 ^4^	1	18 ^4^	1	4 ^4^

Values are means from (a) the nearest BoM weather station, Busselton Aero ^1^, with LTAs over 74 years; (b) the DPIRD weather station, Channybearup ^2^, with LTAs over 10 years; (c) data from the on-farm weather station at Yalyallup ^3^ with LTAs over 12 years from the DPIRD weather station, Capel (CL001) ^4^. * Values in blue font are for <1 °C cooler than the LTA for that month and values in red font indicate values that are >1 °C warmer than the LTA for that month.

## Data Availability

The data presented in this study are openly available from the corresponding authors (DC and SV) and are stored at the University of Western Australia Data Repository (DOI: 10.26182/h98z-yx47).
